# Cross-Talk between the *Aeromonas hydrophila* Type III Secretion System and Lateral Flagella System

**DOI:** 10.3389/fmicb.2016.01434

**Published:** 2016-09-07

**Authors:** Yu-Hang Zhao, Jonathan G. Shaw

**Affiliations:** Department of Infection, Immunity and Cardiovascular Disease, University of SheffieldSheffield, UK

**Keywords:** *Aeromonas*, swarming, motility, lateral flagella, T3SS

## Abstract

*Aeromonas hydrophila* is responsible for aeromonad septicaemia in fish, and gastroenteritis and wound infections in humans. The type III secretion system (T3SS) is utilized by aeromonads to inject protein effectors directly into host cells. One of the major genetic regulators of the T3SS in several bacterial species is the AraC-like protein ExsA. Previous studies have suggested a link between T3SS regulation and lateral flagella expression. The aim of this study was to determine the genetic regulation of the T3SS and its potential interaction with the lateral flagella system in *A. hydrophila*. To investigate the genes encoding the T3SS regulatory components *exsA, exsD, exsC*, and *exsE* were mutated and the activities of the T3SS promoters were measured in wild type and mutant backgrounds demonstrating a regulatory network. The Exs proteins were shown to interact with each other by BACTH assay and Far-Western Blot. The findings suggested a regulatory cascade in which ExsE was bound to the chaperone protein ExsC. When ExsC was free it sequestered the anti-activator ExsD thus stopping the inhibition of the T3SS master regulator ExsA allowing T3SS expression. The T3SS regulatory components were also shown to affect the expression of the lateral flagella system. The activities of the lateral flagella promoters were shown to be repressed by the absence of ExsD and ExsE, suggesting that the T3SS master regulator ExsA was a negative regulator of the lateral flagella system.

## Introduction

*Aeromonas* species are ubiquitous water-borne Gram-negative bacteria that are able to cause a variety of diseases in poikilothermic animals and humans. The psychrophilic *Aeromonas* species represented by *A. salmonicida* predominantly cause furunculosis in salmonid fish as well as many other fish species (McCarthy and Rawle, 1975; Cipriano and Austin, 2011). Human infections are caused by mesophilic aeromonads, of which *A. hydrophila, A. caviae*, and *A. veronii* biovar sobria account for 85% of clinical isolates (Parker and Shaw, 2011). Human infections by aeromonads often result in gastrointestinal disease and wound infection, while septicaemia less commonly occurs in the immunocompromised host (Janda and Abbott, 2010).

The pathogenicity of *Aeromonas* species is associated with a variety of virulence factors such as lipopolysaccharide, S-Layer, enterotoxins, the polar and lateral flagella systems as well as secretion systems (Lowry et al., 2014). The colonization approaches that involve the presence of flagella systems are swimming and swarming (Harshey, 2003). Aeromonads swim using a single polar flagellum, but approximately 60% of motile *Aeromonas* strains possess a second lateral flagella system for swarming motility (Gavin et al., 2002; Kirov et al., 2002). The lateral flagella system in *A. hydrophila* was also found to be involved in host cell adherence and biofilm formation (Gavin et al., 2002; Kirov, 2003).

In *A. hydrophila* AH-3, 38 genes of the lateral flagella system are categorized into nine putative operons, expression of which is controlled in a hierarchal fashion (Wilhelms et al., 2013).

Evidence in *A. hydrophila* AH-1 has shown that the expression level of the two tandem lateral flagellin genes *lafA1 and lafA2* are significantly reduced in *exsD* and *aopN* mutants, genes that are associated with the Type III secretion system (T3SS). However, Δ*exsA* Δ*exsD* and the Δ*exsA* Δ*aopN* double mutations restored the secretion level of the LafA flagellin protein (Yu et al., 2007). This finding indicated potential cross-talk between the lateral flagella system and the T3SS in *A. hydrophila*.

Type III secretion systems (T3SS) have been reported in more than 25 species of Gram-negative bacteria and they are able to inject protein effectors across the plasma membrane directly into the host cell cytosol or to secrete pore-forming translocators that helps effector proteins to get through (Cornelis and Van Gijsegem, 2000; Abby and Rocha, 2012). In *Aeromonas* the T3SS is similar to those reported in *Pseudomonas aeruginosa* and *Yersinia* spp. (Vilches et al., 2004).

The assembly of the T3SS requires a high level of energy and resources; thus it is tightly regulated. In *P. aeruginosa* the master regulator of the T3SS is the AraC family protein ExsA. Transcriptional activation of *exsA* was required for the expression of the T3SS secretion apparatus, translocation machinery and secreted effectors (Hovey and Frank, 1995; Yahr et al., 1995). Moreover, ExsA in *A. hydrophila* shares 76% amino acid homology with ExsA in *P. aeruginosa* (Frank and Iglewski, 1991; Yahr and Frank, 1994; Vilches et al., 2009). As the role of ExsA in *P. aeruginosa* is defined as a transcriptional activator by facilitating RNAP-σ^70^ binding onto the T3SS promoter regions, the role of ExsA in *A. hydrophila* is proposed to be similar.

In *P. aeruginosa* the T3SS master regulator ExsA is controlled by a regulatory cascade of proteins ExsD, ExsC, and ExsE (McCaw et al., 2002; Dasgupta et al., 2004; Rietsch et al., 2005; Urbanowski et al., 2005; Zheng et al., 2007). ExsD is reported to be an anti-activator protein that negatively regulates the T3SS master regulator ExsA through direct protein-protein interaction (McCaw et al., 2002). ExsC is an anti-anti-activator protein that binds to ExsD and prevents it from inhibiting ExsA (Dasgupta et al., 2004). ExsC is the chaperone protein for the secreted protein ExsE, which is found to be a negative regulator of the T3SS (Rietsch et al., 2005). ExsE negatively regulates the T3SS via its physical interactions with ExsC, preventing ExsC from binding to ExsD, thus allowing ExsD to be free to bind and inhibit the transcriptional activation from ExsA (Urbanowski et al., 2005).

In comparison with *P. aeruginosa*, the regulation of the T3SS has not been intensively studied in *A. hydrophila*. Vilches et al. (2004), the complete T3SS region of *A. hydrophila* was sequenced, but the regulation of the T3SS in *A. hydrophila* still largely unknown. The ExsA and ExsD proteins in *A. hydrophila* SSU strain had similar effects on the regulation of the T3SS to those found in *P. aeruginosa* using mutagenesis and over-expression assays (Sha et al., 2007). In *A. hydrophila* AH-3, quantitative RT-PCR results for *aopN* and *aexT* mRNA production showed 60–85% decrease in a Δ*exsA* mutant background and the promoters activities *aopN* and *aexT* genes were also significantly decreased (Vilches et al., 2009). All these findings suggest that ExsA, like its homologue in *P. aeruginosa*, is likely to be the master regulator of the T3SS in *A. hydrophila*. However, there is little evidence of ExsC and ExsE involvement in the regulation of the T3SS in *Aeromonas* species. In this study we demonstrate the presence of a T3SS regulatory cascade in *A. hydrophila* and provide further evidence for the cross-talk between the T3SS regulators and expression of the lateral flagella system.

## Materials and Methods

### Media, Bacterial Strains, Plasmids, Primers and Antibiotics

The bacterial strains used in this study are listed in **Table 1**. Plasmids used in this study are listed in **Table 2**. *Escherichia coli* strains were grown on Luria Bertani (LB) Miller agar and in LB Miller broth, while *A. hydrophila* strains were grown on Columbia Blood Agar (Oxoid, Basingstoke, UK) and in LB Miller broth. *A. hydrophila* strains were incubated at 30°C while *E. coli* strains were incubated at 37°C. Kanamycin [Km] (50 μg/ml), streptomycin [Sm] (50 μg/ml), ampicillin [Amp] (100 μg/ml), rifampicin [Rif] (50 μg/ml), chloramphenicol [Cm] (50 μg/ml) and gentamycin [Gm] (50 μg/ml) were added when required. Primers used in this study are listed in Supplementary Table S1.

**Table 1:** Bacterial strains used in this study.

Name of strains	Description	Reference
***Aeromonas hydrophila* strains**		
AH3R	Wild type *Aeromonas hydrophila* AH3 strain, *Rif^R^*	Dr. J. Tomás University of Barcelona
A5	AHR strain with miniTn5Km insertion in *exsD*, Km^R^	This study
A25	AHR strain with miniTn5Km insertion in *exsD*, Km^R^	This study
*exsA* mutant	AH3R strain with *exsA* knocked out by the insertion of Km^R^ cassette	This study
*exsC* mutant	AH3R strain with *exsC* knocked out by the insertion of Km^R^ cassette	This study
*exsD* mutant	AH3R strain with *exsD* knocked out by the insertion of Km^R^ cassette	This study
*exsE* mutant	AH3R strain with *exsE* knocked out by the insertion of Km^R^ cassette	This study
*lafK* mutant	AH3R strain with *lafK* knocked out by the insertion of Km^R^	This study
AH3R-*PascN*	Reporter plasmid pKAG-P*ascN* conjugated into AH3R wild type	This study
AH3R-*PexsA*	Reporter plasmid pKAG-P*exsA* conjugated into AH3R wild type	This study
AH3R-*PexsD*	Reporter plasmid pKAG-P*exsD* conjugated into AH3R wild type	This study
AH3R-*PfliM*	Reporter plasmid pKAG-P*fliM* conjugated into AH3R wild type	This study
AH3R-P*lafK*	Reporter plasmid pKAG-P*lafK* conjugated into AH3R wild type	This study
AH3R-P*flgM*	Reporter plasmid pKAG-P*flgM* conjugated into AH3R wild type	This study
AH3R-P*flgA*	Reporter plasmid pKAG-P*flgA* conjugated into AH3R wild type	This study
AH3R-P*flgB*	Reporter plasmid pKAG-P*flgB* conjugated into AH3R wild type	This study
AH3R-P*maf*	Reporter plasmid pKAG-P*maf* conjugated into AH3R wild type	This study
AH3R-P*lafA*	Reporter plasmid pKAG-P*lafA* conjugated into AH3R wild type	This study
AH3R-P*lafB*	Reporter plasmid pKAG-P*lafB* conjugated into AH3R wild type	This study
AH3R-P*lafX*	Reporter plasmid pKAG-P*lafX* conjugated into AH3R wild type	This study
***Escherichia coli* strains**		
*DH5α*	F-, Φ80*lac*ZΔM15, Δ(*lac*ZYA-*arg*F), U169, *rec*A1, *end*A1, *hsd*R17, (rK-, mK+), *pho*A, *sup*E44, λ-*thi*-1, *gyr*A96, *rel*A1	Hanahan, 1983
CC118-λ*pir*	*phoA20, thi-1, rspE, rpoB, argE*, (Am), *recA1*, phage λ*pir*	Herrero et al., 1990
S17-1-λ*pir*	Sm^R^, F-, *recA, hsdR*, RP4-2 (Tc::Mu) (Km::Tn*7*), phage λ*pir*	Miller, 1972
Sm10-λ*pir*	KmR, *thi*-1, *thr, leu, tonA, lacY, supE, recA*::RP4-2-Tc::Mu, phage λ*pir*	Miller and Mekalanos, 1988
HB101	F^-^ *mcrB mrr hsd*S20(r_B_^-^ m_B_^-^) *recA*13 *leuB*6 *ara*-14 *proA*2 *lacY*1 *galK*2 *xyl*-5 *mtl*-1 *rpsL*20(Sm^R^) *gln*V44 λ^-^	Hanahan, 1983
BL21(DE3)	F^-^ *omp*T *gal dcm lon hsdS*_B_(r_B_^-^ m_B_^-^) λ(DE3)	Novagen
C41(DE3)	*ompT hsdSB (rB- mB-) gal dcm* (DE3)	Lucigen
BL21Star^TM^(DE3)	F^-^ *omp*T *hsd*S_B_(r_B_^-^, m_B_^-^) *gal dcm rne*131 (DE3)	Invitrogen
BTH101	F^-^, *cya*-99, *araD*139, *galE*15, *galK*16, *rpsL*1(*Str*^R^), *hsdR*2, *mcrA1, mcrB1*	Euromedex
*E. coli* ER2523 (NEB Express)	*fhuA2 [lon] ompT gal sulA11 R(mcr-73::miniTn10–*TetS*)2 [dcm] R(zgb-210::Tn10–*TetS*) endA1*Δ*(mcrC-mrr)114::IS10*	New England BioLabs

**Table 2:** Plasmids used in this study.

Name of plasmids	Characteristics	Reference
pBBR1MCS	Broad-host-range plasmid, Cm^R^	Kovach et al., 1994
pBBR1MCS-5	Broad-host-range plasmid, Gm*^R^*	Kovach et al., 1994
pBB*exsCEB*	*exsCEB* region cloned into pBBR1MCS	This study
pBB*exsCKm^R^*	*exsC::*Km^R^ knockout ligated with pBBR1MCS	This study
pBBR5*exsA*	*exsA* ligated with pBBR1MCS-5	This study
pUTmini-Tn5Km1	Suicide vector carrying Tn5, Amp^R^ Km^R^	de Lorenzo et al., 1990
pUC4KIXX	Source of Km^R^ cassette *(npt*II)	Amersham Pharmacia
pJMK30	pUC19 derivative, containing a Km^R^ cassette (AphA-3) from *Campylobacter coli*	van Vliet et al., 1998
pKNG101	RK6 derived suicide plasmid, *SacB*, Sm^R^	Kaniga et al., 1991
pKNG*exsA*Km^R^	*exsA*::Km^R^ knockout ligated with pKNG101 suicide plasmid	This study
pKNG*exsC*Km^R^	*exsC*::Km^R^ knockout ligated with pKNG101 suicide plasmid	This study
pKNG*exsD*Km^R^	*exsD*::Km^R^ knockout ligated with pKNG101 suicide plasmid	This study
pKNG*exsE*Km^R^	*exsE*::Km^R^ knockout ligated with pKNG101 suicide plasmid	This study
pGEM-3Zf(+)	Cloning vector, used for ISA-cloning in this study, Amp^R^	Promega
pET28a	Over-expression vector, T7 promoter, N-terminal His tag, Km^R^	Novagen
pET28*exsA*	*exsA* ligated in frame with His-tag in pET28a	This study
pET28*exsC*	*exsC* ligated in frame with His-tag in pET28a	This study
pET28*exsD*	*exsD* ligated in frame with His-tag in pET28a	This study
pET28*exsE*	*exsE* ligated in frame with His-tag in pET28a	This study
pMAL-c5x	Over-expression vector, *malE*-, MBP5 coding, Amp^R^	New England BioLabs
pMAL*exsA*	*exsA* ligated in frame with MBP5 gene in pMAL-c5x	This study
pMAL*exsC*	*exsC* ligated in frame with MBP5 gene in pMAL-c5x	This study
pMAL*exsD*	*exsD* ligated in frame with MBP5 gene in pMAL-c5x	This study
pMAL*exsE*	*exsE* ligated in frame with MBP5 gene in pMAL-c5x	This study
pKAGb-2(-)	Broad-host-range reporter vector, ori_1600_ carrying promoter-less *lacZ* gene, Cm^R^	Tabei et al., 2009
pKAG-P*ascN*	Promoter upstream of *ascN* cloned into pKAG-b2(-)	This study
pKAG-P*aopN*	Promoter upstream of *aopN* cloned into pKAG-b2(-)	This study
pKAG-P*exsC*	Promoter upstream of *exsC* cloned into pKAG-b2(-)	This study
pKAG-P*exsA*	Promoter upstream of *exsA* cloned into pKAG-b2(-)	This study
pKAG-P*exsD*	Promoter upstream of *exsD* cloned into pKAG-b2(-)	This study
pKAG-P*fliM*	Promoter upstream of *fliM* cloned into pKAG-b2(-)	This study
pKAG-P*flgB*	Promoter upstream of *flgB* cloned into pKAG-b2(-)	This study
pKAG-P*lafA*	Promoter upstream of *lafA* cloned into pKAG-b2(-)	This study
pKT25	pSU40 derivative, *lac* promoter, allow in-frame fusion at C-terminal of T25, Km^R^	Euromedex
pKNT25	pSU40 derivative, *lac* promoter, allow in-frame fusion at N-terminal of T25, Km^R^	Euromedex
pUT18	pUC19 derivative, *lac* promoter, allow in-frame fusion at N-terminal of T18, Amp^R^	Euromedex
pUT18C	pUC19 derivative, *lac* promoter, allow in-frame fusion at C-terminal of T18, Amp^R^	Euromedex
pKT25-zip	pKT25 derivative, leucine zipper of GCN4 fused at C-terminal of T25, Km^R^	Euromedex
pUT18C-zip	pUT18C derivative, leucine zipper of GCN4 fused at C-terminal of T25, Km^R^	Euromedex
pKT25-*exsA*	*exsA* cloned in-frame with pKT25	This study
pKT25-*exsC*	*exsC* cloned in-frame with pKT25	This study
pKT25-*exsD*	*exsD* cloned in-frame with pKT25	This study
pKT25-*exsE*	*exsE* cloned in-frame with pKT25	This study
pKNT25-*exsA*	*exsA* cloned in-frame with pKNT25	This study
pKNT25-*exsC*	*exsC* cloned in-frame with pKNT25	This study
pKNT25-*exsD*	*exsD* cloned in-frame with pKNT25	This study
pKNT25-*exsE*	*exsE* cloned in-frame with pKNT25	This study
pUT18-*exsA*	*exsA* cloned in-frame with pUT18	This study
pUT18-*exsC*	*exsC* cloned in-frame with pUT18	This study
pUT18-*exsD*	*exsD* cloned in-frame with pUT18	This study
pUT18-*exsE*	*exsE* cloned in-frame with pUT18	This study
pUT18C-*exsA*	*exsA* cloned in-frame with pUT18C	This study
pUT18C-*exsC*	*exsC* cloned in-frame with pUT18C	This study
pUT18C-*exsD*	*exsD* cloned in-frame with pUT18C	This study
pUT18C-*exsE*	*exsE* cloned in-frame with pUT18C	This study

### DNA Techniques

General DNA manipulation methods involving DNA restriction endonuclease, T4 DNA ligase and alkaline phosphatase were used as recommended by the supplier (New England Biolabs) to construct plasmids in this study, including pKNG101 constructs, pET28a constructs, pMAL-c5x constructs, pKAGb-2(-) constructs, pKT25 constructs, pKNT25 constructs, pUT18 constructs and pUT18C constructs. PCR amplification was performed using Platinum Pfx DNA polymerase (Invitrogen) while PCR screening was performed using Taq DNA polymerase (New England Biolabs).

### Mini-Tn5Km1 Mutagenesis

Conjugal transfer of pUT-mini-Tn5Km from *E. coli* S17-1λ*pir* to *A. hydrophila* AH-3R was performed using a filter mating technique. Bacterial conjugation was allowed to proceed for 6–8 h at 30°C on sterile nitrocellulose filters (0.45 mm pore size) placed onto a Blood agar plate. Serial dilutions of the mating mix were plated on LB agar supplemented with Rif and Km, the latter added in order to select for mini-Tn5Km. All mutants were then analyzed for the presence of the transposon by Southern hybridisations of *Pst*I chromosomal DNA digestions, as described before (Gryllos et al., 2001). As no *Pst*I restriction sites were present in the transposon, variable size bands larger than the transposon were observed for each mutant. To determine the site of the transposon insertion, chromosomal DNA was isolated and direct genomic sequencing was employed using 24-mer primers were designed to the O and the I end of transposon Tn5 and a 99 cycle polymerase sequencing reaction

#### Nucleotide Sequencing and Sequence Analysis

Double-stranded DNA sequencing was performed by using the Sanger dideoxy-chain termination method with the Abi Prism dye terminator cycle sequencing kit (Perkin Elmer). DNA fragments were ligated into appropriate primers and sequenced using an ABI PRISM 377 DNA sequencer (Perkin-Elmer Corporation). Universal or custom designed primers were employed in sequencing the ends of the DNA inserts (**Table 3**). Following the first sequencing reaction and whenever required, primers were designed until the inserts’ sequences were complete. Primers used for DNA sequencing were purchased from Eurogentec. For chromosomal walking to extend the sequence into flanking regions direct genomic sequencing was used. Custom 24-mer primers were designed to a known nucleotide sequence of the O and the I end of trasposon Tn5 and were used with sheared *A. hydrophila* genomic DNA in a 99 cycle polymerase reaction using the BigDye terminator mix according to the manufacturer’s instructions (P.E.-Applied Biosystems).

**Table 3:** Investigation of the interactions between each two of the aeromonad Exs proteins using BACTH system.

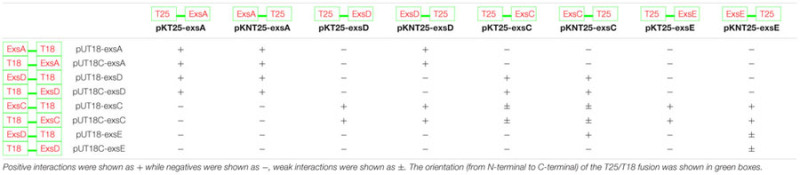

### Construction of Defined Insertional *exsA, exsC* and *lafK* Mutants

In order to facilitate a knockout in the *exsA, exsC* or *lafK* genes, a selectable marker was inserted in the middle of the gene. Mutants were created by the insertion of the Tn5 derived kanamycin resistance cartridge (*nptII*) from pUC4-KIXX (Amersham). This cartridge contains an outward reading promoter that drives the transcription of downstream genes when inserted in the correct orientation. Although the cartridge insertions ensure transcription of downstream genes, the regulation and expression levels of these genes could be altered. For each mutant the 1.4 kb *Sma*I digested kanamycin resistance cartridge was inserted into a convenient site within the middle of the gene. If a convenient site was not present, one was created by PCR. Constructs containing the mutated genes were ligated into the suicide vector pKNG101 and transferred into *Aeromonas* by conjugation to allow allelic exchange as described before (Tabei et al., 2009). After conjugation, a double crossover event was selected by picking colonies that were streptomycin sensitive and kanamycin resistant. The potential mutants were checked by PCR screening using the appropriate amplification primers followed by sequencing (provided by Core Genomic Facility, University of Sheffield).

### Construction of *exsD* and *exsE* Mutant Using Isothermal Assembly (ISA)

Isothermal assembly was used to assemble multiple DNA fragments into one plasmid vector in a single reaction (Gibson et al., 2009). ISA required three DNA fragments and one digested plasmid for the assembly, in which *exsD* was amplified by PCR into two fragments using primers *exsD* F1 forward/reverse and *exsD* F2 forward/reverse (**Table 3**), each containing adapter sequence overlapping with the plasmid or the kanamycin cassette. The kanamycin cassette was amplified by PCR from plasmid pJMK30 using Pfx DNA polymerase and primers Kan forward/reverse (**Table 3**), while the plasmid pGEM-3Zf(+) was digested with the restriction enzyme *Hinc*II. Each fragment assembled in this reaction should have an approximately 30 bp adapter sequence overlapping each other. Then the fragments, which were equi-molar when added, were mixed together with ISA Buffer (Gibson et al., 2009) and three enzymes: T5 exonuclease, Phusion polymerase and Taq ligase. This mixture was incubated at 50°C overnight and transformed into *E. coli* DH5α to select for ampicillin and kanamycin resistance, generating pGEM*exsD*Km^R^. The pGEM*exsE*Km^R^ plasmid construct was assembled using the same method. Both constructs were checked by nucleotide sequencing. The *exsD*::Km^R^ fragment was then amplified from the pGEM*exsD*Km^R^ by PCR using Pfx DNA polymerase with *exsD*_pGEM forward and *exsD*_pGEM reverse primers (**Table 3**). A similar PCR amplification was carried out to obtain the *exsE::*Km^R^ fragment (*exsE*_pGEM forward and *exsE*_pGEM reverse primers) on the plasmid construct pGEM*exsE*Km^R^. Both PCR fragments of *exsD*::Km^R^ and *exsE*::Km^R^ had blunt ends and were ligated into *Sma*I-digested suicide plasmid pKNG101. After ligation, the samples were transformed into competent cells of *E. coli* CC118-λ*pir* to select for kanamycin and streptomycin resistant colonies, containing pKNG*exsD*Km^R^ or pKNG*exsE*Km^R^ constructs. Once the suicide plasmid constructs were prepared, they were transformed into the *E. coli* donor cell S17-1-λ*pir* and then conjugated into *A. hydrophila* AH3R to allow allelic exchange. A double crossover event was selected by picking up colonies that were streptomycin sensitive and kanamycin resistant. The chromosomal DNA of these potential *exsD* or *exsE* mutants were then extracted and used as templates for PCR screening using Taq DNA polymerase with *exsD* F1 forward and *exsD* F2 reverse primers, followed by sequencing to confirm the knockout. The same PCR screening and sequencing were carried out on potential *exsE* mutant using *exsE* F1 forward and *exsE* F2 reverse primers.

### Swarming Assay

Swarming motility of *A. hydrophila* strains were assessed on swarming agar plates (0.5% NaCl, 0.6% Difco Nutrient Broth, and 0.6% Eiken agar). A 1ml volume of the bacterial strains that were pre-cultured in broth media were centrifuged at 15,000 × *g* for 1min. A sterile toothpick was used to transfer a small amount (less than a colony size) of the pellet into the center of swarming agar plates. The plates were incubated face-up at 30°C overnight. Swarming motility was measured by examining the migration of bacteria across the agar (swarming diameters) from the center toward the periphery of the plate (Kirov et al., 2004). Bacterial cells required for lateral flagella transcriptional fusion assays were grown at 30°C overnight and the edge of the bacterial swarm was resuspended in sterile PBS for the β-galactosidase assay.

### β-Galactosidase Assay

The promoter of interest was fused to the promoter-less *lacZ* on the reporter plasmids pKAGb-2(-), followed by conjugation into *A. hydrophila* mutant and wild type strains as described elsewhere (Tabei et al., 2009). Activity of the promoters were measured as a function of β-galactosidase activity. *A. hydrophila* cultures were grown in triplicate to an OD600 of 0.5 – 0.8 and were then chilled on ice for 15 min. Duplicate assays were performed at 30°C on 200 μl of cells for each culture in a total volume of 1ml following permeabilization of the cells with chloroform sodium-dodecyl sulfate (Miller, 1972). Values are presented in Miller units (MU).

### Protein Over-Expression and Purification

The *E. coli* protein expression strain (BL21Star or NEB ER2523 Express) containing the plasmid of interest (pET28a or pMAL-c5x constructs respectively) was incubated overnight in 10 ml LB broth with appropriate antibiotics and 1% (w/v) filter sterilized glucose. On the next day, 1 ml of the culture was transferred into 1 L of LB broth with appropriate antibiotics and 1% (w/v) filter-sterilized glucose. It was then incubated at 37°C for approximately 2 h until the OD_600_ was around 0.2. At this point, 0.3 mM – 1 mM IPTG was added to induce the protein expression. After 2–3 h incubation, the cells were harvested by centrifugation at 8,000 × *g* for 15 min at 4°C. The pellet was resuspended in Binding Buffer [10 mM Imidazole, 500 mM NaCl, 5% (v/v) Glycerol and 20 mM Phosphate Buffer] for Histidine-tagged proteins or in Column Buffer (20 mM Tris-HCl pH7.4, 200 mM NaCl, 1 mM EDTA, 1 mM sodium azide and 10 mM β-mercaptoethanol) for MBP-tagged proteins and frozen overnight at -20°C. The pellet was then sonicated using Jencons Vibracell at 20 kHz (20% amplitude) for 8x 20 s with 1 min intervals on ice. The sonicated sample was then centrifuged at 30,000 × *g* for 30 min at 4°C to separate soluble and insoluble proteins. After sonication, the supernatant that contained soluble proteins was transferred into a fresh universal tube while the pellet was stored at -20°C. Histidine-tagged proteins (pET28a constructs) were purified using 1 ml HisTrap HP Column (GE Healthcare) as recommended by the supplier. While MBP-tagged proteins (pMAL-c5x constructs) were purified using 1ml MBPTrap HP columns (GE Healthcare) according to the pMAL Protein Fusion and Purification System manual (New England BioLabs). Purification to approximately 95% was assessed by SDS-PAGE.

### Western Blot and Far-Western Blot

Proteins were separated by SDS-PAGE and transferred onto a nitrocellulose membrane (0.45 μm pore size). The membrane was then blocked in 20 ml of PBS + 5% (w/v) dry skimmed milk powder for 1 h. After blocking, the membrane was washed 10 min in 20 ml PBS Buffer with 0.1% (v/v) Tween20 (PBST). Then 10 μl (1:2000) of primary antibody (anti-penta-His [Qiagen] for His-tagged proteins and anti-MBP [New England BioLabs] for MBP-tagged proteins) was added to 20 ml of PBST + 5% milk, in which the membrane was soaked for 1 h. After primary antibody binding, the membrane was washed three times 10 min in 20 ml PBST Buffer. Then the membrane was probed with 20 ml PBST + 5% milk with 5 μl HRP-conjugated secondary antibody (1:4000) for 1 h. After secondary antibody binding, the membrane was washed three times 10 min in 20 ml PBST Buffer again and was developed by Pierce ECL Western Blotting and Chemidoc XRS+ System (Bio-Rad).

Far-Western blotting was based on western blotting and used to investigate protein–protein interactions. In Far-Western Blots, the target proteins were probed with a putative interacting protein, which had a different tag to the target protein. The interactions between two proteins could then be detected by Western Blot using antibodies against the tag on the putative interacting protein. After the transfer of proteins and blocking, the nitrocellulose membrane was probed with a non-antibody protein with a different fusion tag to the loaded proteins. The membrane was probed for 1 h followed by three times 10 min wash with PBST Buffer. Then the membrane was then incubated with the primary antibody against the tag on the probing protein for 1 h followed by three times 10 min wash with PBST Buffer. This was followed by a conjugated secondary antibody against the primary antibody and following washing the membrane was developed as above.

### Bacterial Adenylate Cyclase Two-Hybrid (BACTH) Assay

The BACTH Assay was carried out using the Euromedex BACTH System Kit. The target genes were first cloned into the BACTH plasmids (pKT25, pKNT25, pUT18 and pUT18C) in frame with either T25 or T18 fragments of the *cyaA* gene at the 5′ or 3′ end of the gene of interest (**Table 2**), to allow co-expression of fusion proteins. Then one of the T25 derived plasmid constructs was co-transformed with a T18 derived plasmid construct into *E. coli* BTH101 reporter cells and incubated on MacConkey/maltose agar [4% (w/v) MacConkey agar base (Difco^TM^), 1% (w/v) maltose, 0.5mM IPTG and appropriate antibiotics) for 2 nights at 30°C.

When the two proteins of interest interacted with each other, heterodimerization of the fusion proteins allowed the complementation of the T25 and T18 fragments to form a catalytic domain of adenylate cyclase (CyaA), thus cAMP was synthesized. Then the *mal* operon was activated by cAMP/CAP complex. Therefore the maltose metabolism pathway was switched on in the *E. coli* BTH101 reporter strain, which allowed the fermentation of maltose and the production of acid that turned the pH indicator in MacConkey agar pink. Therefore positive interaction colonies were pink/red in color whereas negative colonies were white.

## Results

### Mutation of ExsD Affects Swarming But Not Swimming Motility in *A. hydrophila*

In order to isolate mutants involved in swarming motility that were encoded outside the lateral flagella gene cluster, random transposon mutagenesis was performed. Over 2000 mutants generated by this method were qualitatively screened for reduced or increased swarming on swarm plates. Several mutant strains consistently exhibited reduced swarming when compared to the wild type, however, their swimming ability in motility agar remained the same as the wild type. In two of the mutants (A5 and A25) the transposon had inserted into 3′ end of the *exsD* gene of the aeromonad type III secretion system (T3SS). As the aeromonad T3SS is very similar to that found in *P. aeruginosa* (Vilches et al., 2004) the aeromonad *exsD* gene encodes a protein that is the anti-activator of ExsA the major transcriptional regulator of the T3SS.

### Exs Proteins Modulate T3SS Promoter Activity

The regulator ExsA usually works as an activator of T3SS although there is some evidence that it can act as a repressor of other systems such flagella. In order to test whether the mutations in the *exsD* gene are allowing ExsA to act directly or indirectly as a repressor we created individual mutations in the putative regulator and anti-activator genes of the aeromonad T3SS system, namely *exsA, exsD, exsC*, and *exsE*.

**FIGURE 1:**
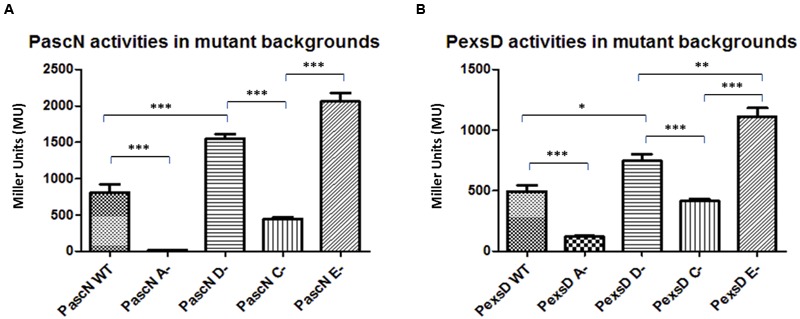
**β-galactosidase activities of promoters *PascN***(A)** and *PexsD***(B)** in *A. hydrophila* AH3R wild type and mutant backgrounds.** The promoter activities were measured when each strain was grown in LB broth to log phase at 30°C with shaking at 200 rpm. The promoter activities of *PascN* and *PexsD* were significantly decreased in the *exsA* (A-) mutant and *exsC* (C-) mutant when compared to the promoter activities in *exsD* (D-) mutant and *exsE* (E-) mutant (*p* < 0.001). Also, the promoter activities in the wild type strain were significantly lower than both the *exsD* mutant and the *exsE* mutant strains (*p* < 0.001). Assays were carried out in triplicate and the values are the means. The error bars showed Standard Error of the Mean (SEM). The graph was created using GraphPad Prism 5. The significance was determined using Student’s *t*-test (^∗^*p* < 0.05, ^∗∗^*p* < 0.01, ^∗∗∗^*p* < 0.001).

Firstly to investigate the role of the genes in the aeromonad T3SS, the promoter regions of the T3SS operons (Vilches et al., 2004, 2009) were isolated and fused to the promoter-less *lacZ* gene in the transcriptional reporter plasmid pKAGb-2(-). The promoter activities of a selection of the promoter constructs were then investigated in the *A. hydrophila* wild type and *exsA, exsC, exsD* and *exsE* mutant strains. The individual reporter plasmids were introduced into the *A. hydrophila* strains and the promoter activities measured. The activity of promoter *PascN* in the *A. hydrophila* wild type was 808 MU, this was reduced in the *exsA* mutant, in which the promoter activity was down to 15 MU (**Figure 1A**). In the *exsD* mutant, the PascN promoter activity increased to 1551 MU, which was an increase from the activities in both wild type and *exsA* mutant strains (*p* < 0.001). The *PascN* promoter activity was repressed, but not completely shut down, to 444 MU in the *A. hydrophila exsC* mutant (**Figure 1A**). While in the *A. hydrophila exsE* mutant strain, the promoter activity was further increased to 2059 MU, which was a significant increase compared to the activities in the *A. hydrophila exsA* and *exsC* mutants (*p* < 0.001).

The promoter *PexsD* showed a similar trend of activities as for the *PascN* promoter. In the *A. hydrophila* wild type strain *PexsD* activity was 494 MU and it was significantly reduced to 121 MU when *exsA* gene was knocked out (*p* < 0.001) (**Figure 1B**). In the *A. hydrophila exsD* mutant, the promoter activity of *PexsD* increased to 744 MU, which was higher than the promoter activity measured in the wild type strain (*p* < 0.05). While in the *exsC* mutant the promoter activity was reduced to 413 MU, which was close to the wild type value but significantly lower than in the *exsD* mutant strain (*p* < 0.001). When *exsE* was absent, the promoter activity of *PexsD* reached 1109 MU, which was a significant increase compared to the activities in all other backgrounds (*p* < 0.01) (**Figure 1B**). The patterns of the promoter activities in the different mutant backgrounds suggest a modulating regulatory cascade similar to that of *P. aeruginosa* (Diaz et al., 2011).

**FIGURE 2:**
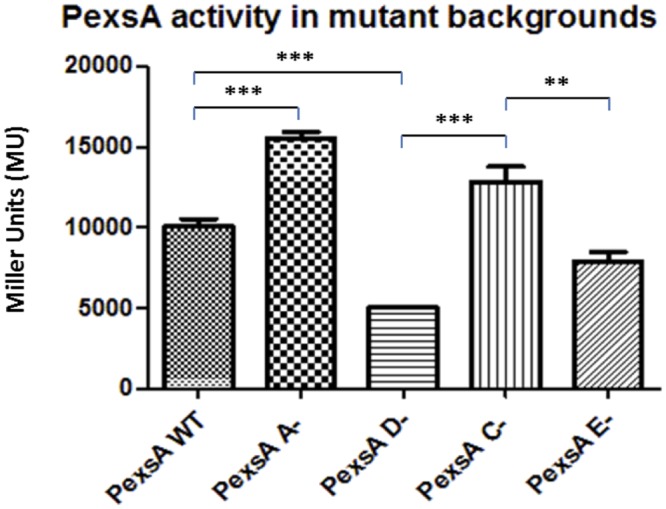
**β-galactosidase activities of promoter *PexsA* in *A. hydrophila* AH3R wild type and mutant backgrounds.** The promoter activity of *PexsA* demonstrated a significant increase in the *exsA* (A-) mutant and *exsC* (C-) mutant backgrounds when compared to the promoter activities measured in the *exsD* (D-) mutant and *exsE* (E-) mutant backgrounds (*p* < 0.01). Assays were carried out in triplicate and the values are the means. The error bars showed Standard Error of the Mean (SEM). The significance was determined by Student’s *t*-test (^∗∗^*p* < 0.01, ^∗∗∗^*p* < 0.001).

The promoter *PexsA*, which was responsible for the expression of the putative T3SS master regulator ExsA in *A. hydrophila*, the promoter activity was much higher than the other promoters of the T3SS. In the wild type the *PexsA* promoter activity was 10,066 MU. Unlike the other promoters of the T3SS, all of which were repressed when *exsA* was knocked out, the promoter activity of *PexsA* was not reduced but demonstrated a significant increase in activity in the *exsA* mutant to 15,546 MU (*p* < 0.001) (**Figure 2**). More surprisingly, the promoter activity of *PexsA* decreased to 5,030 MU in the *exsD* mutant background, which was significantly lower than the promoter activities of both the wild type and the *exsA* mutant strains (*p* < 0.001). In the *exsC* mutant, the promoter activity of *PexsA* increased to 12,826 MU, which was significantly higher than both the activities in the wild type (*p* < 0.05) and in the *exsD* mutant strains (*p* < 0.001), but not significantly different from the promoter activity in the *exsA* mutant. The promoter activity of *PexsA* in the *exsE* mutant was 7,860 MU, which was significantly lower than both the activities in the *exsA* mutant (*p* < 0.001) and in the *exsD* mutant strains (*p* < 0.01) (**Figure 2**).

### ExsA Negatively Regulates Its Own Promoter

Due to the unexpected results of the *PexsA* promoter activities in the different *A. hydrophila* backgrounds, another approach was required to investigate the regulation of the T3SS. Thereby, *E. coli* was utilized as a heterologous host to re-constitute T3SS regulation by co-transforming two plasmids into this cell background. The reporter plasmid pKAGb-2(-) containing the aeromonad T3SS promoters fused to *lacZ*, and the gene encoding ExsA the putative master regulator of the *A. hydrophila* T3SS, was cloned into the broad host range pBBR1MCS-5 plasmid. Both plasmids were co-transformed into *E. coli* and assayed for β-galactosidase activity.

**FIGURE 3:**
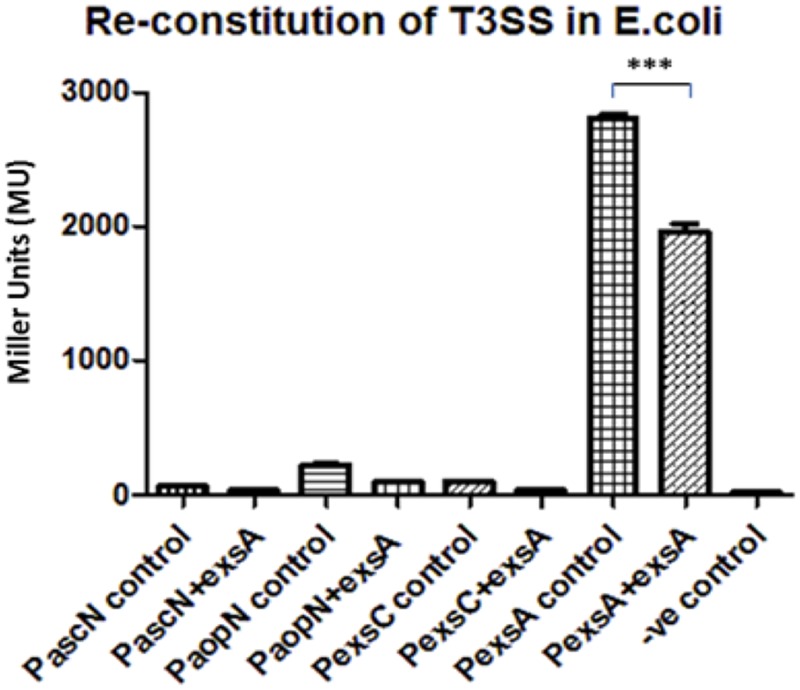
**Re-constitution of T3SS regulation in *E. coli* DH5α.** The promoters (P) of the aeromonad T3SS were cloned into reporter plasmid pKAGb-2(-) and fused upstream of a promoter-less *lacZ* gene. The *A. hydrohphila* AH3R *exsA* gene was cloned into pBBR1MCS-5 plasmid and co-transformed into *E. coli* DH5α with pKAGb-2(-) reporter plasmid constructs. β-galactosidase assays were carried out to measure the promoter activity in the presence or absence of *exsA in-trans*. Each T3SS promoter activity was measured in the presence of *exsA* (+*exsA*) or absence of *exsA* (control). Negative control (-ve) was measured using promoter-less pKAGb-2(-) plasmid. All of the *A. hydrophila* T3SS promoters had very low activities in the *E. coli* re-constitution system except for the promoter *PexsA*. The promoter activity of *PexsA* was significantly higher in the absence of *exsA* rather than in the presence of *exsA in-trans* (*p* < 0.001). Assays were carried out in triplicate and the values are the means. The error bars showed Standard Error of the Mean (SEM). The significance was determined using Student’s *t*-test (^∗∗∗^*p* < 0.001).

Each of the T3SS promoter activities was measured in the presence or absence of *exsA* in *E. coli* cells by co-transforming the reporter plasmid with pBBR5*exsA* or empty pBBR1MCS-5 respectively (**Figure 3**). All of the T3SS promoters, except for promoter *PexsA*, had very low activity in *E. coli*, whether or not *exsA* was present *in-trans*. When the promoter activity of *PexsA* was measured with empty pBBR1MCS-5, the β-galactosidase activity was approximately 2,806 MU, but when measured with pBBR5*exsA* construct, the promoter activity of *PexsA* had decreased to approximately 1,962 MU (*p* < 0.001) (**Figure 3**). This agreed with our findings of *PexsA* promoter activities in *A. hydrophila* that the master regulator ExsA negatively regulates its own promoter.

### Demonstration of ExsA-ExsD, ExsD-ExsC, and ExsC–ExsE Protein–Protein Interactions

**FIGURE 4:**
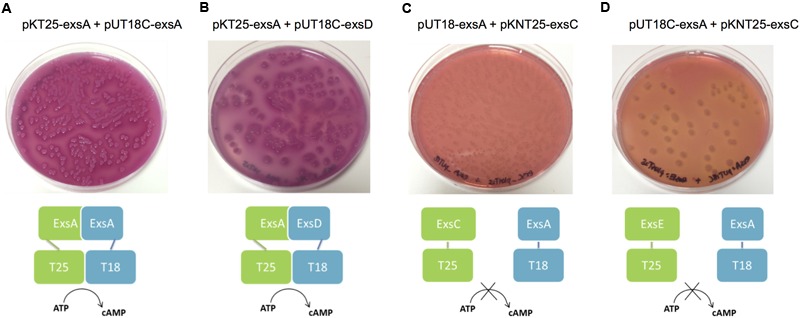
**Illustration of BACTH interactions between ExsA and each of the Exs proteins.** Plasmid combinations were co-transformed into *E. coli* BTH101 and grown on MacConkey-maltose agar for 2 days at 30°C. **(A)** pKT25-*exsA* with pUT18C-*exsA* showing strong ExsA–ExsA self-interaction **(B),** pKT25-*exsA* with pUT18C-*exsD* showing strong ExsA–ExsD interaction; **(C),** pUT18-*exsA* with pKNT25-*exsC* showing no interaction between ExsA and ExsC; **(D),** pUT18C-*exsA* with pKNT25-*exsE* showing no interaction between ExsA and ExsE. The BACTH assay was carried out three times for each combination.

Extrapolation from other related bacterial T3SS indicate the master regulator of the T3SS in the *A. hydrophila* is ExsA (Diaz et al., 2011). This protein is able to self-interact for cooperative activation of the T3SS promoters as well as interacting with the anti-activator ExsD, while not interacting with ExsC or ExsE (Diaz et al., 2011). It is not known if a regulatory cascade similar to that of *P. aeruginosa* occurs in *A. hydrophila*. Therefore a bacterial two-hybrid approach was utilized to investigate the potential protein–protein interactions between the aeromonad Exs proteins. Each *exs* gene was inserted into all four bacterial two-hybrid plasmid vectors. All combinations are shown in **Table 3**. These plasmid combinations were used to co-transform the reporter strain *E. coli* BTH101. ExsA–ExsA interactions were demonstrated with co-transformation of pKT25-*exsA* with either pUT18-*exsA* or pUT18C-*exsA*, and with pKNT25-*exsA* with pUT18-*exsA* or pUT18C-*exsA* (**Figure 4A**; **Table 3**). Each of the *exsA*-fused BACTH plasmid constructs showed positive interactions when co-transformed with each of the *exsD*-fused BACTH plasmid constructs (**Figure 4B**). However, ExsA showed no interaction with ExsC or ExsE (**Figures 4C,D**; **Table 3**).

ExsD which had been shown to interact with ExsA, showed no evidence of ExsD–ExsD self-interaction. However, ExsD was shown to interact with ExsC in every combination, while no evidence of ExsD–ExsE interaction was found (**Table 3**).

ExsC did not interact with ExsA in any combination. Strong ExsC–ExsD interactions were shown in every case, while weak interactions were observed for ExsC–ExsC self-interaction due to weaker colouration than seen with ExsC–ExsD or ExsC–ExsE. Very strong ExsC–ExsE interaction was observed in every combination of *exsC*-fused BACTH plasmid constructs with *exsE*-fused BACTH plasmids (**Table 3**).

ExsE was shown to interact with ExsC only, the *exsE*-fused BACTH plasmids showed no positive interaction with either ExsA or ExsD. As demonstrated in the ExsC interactions, ExsE–ExsC interactions were very strong in every case. A weak ExsE–ExsE self-interaction was observed (**Table 3**).

To confirm the interaction results from the bacterial two-hybrid assay, the Exs proteins were further investigated by Far western blot. In order to facilitate this each protein was expressed and purified as both His-tagged and MBP-fused versions.

**FIGURE 5:**
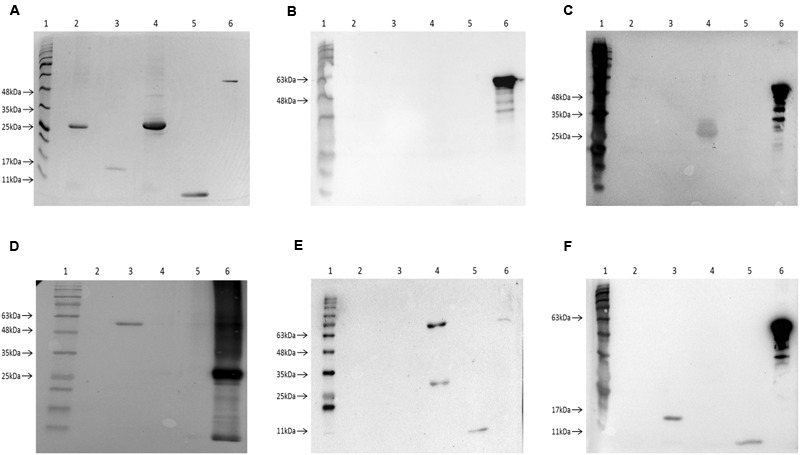
**Investigation of the Exs protein interactions using Far-Western Blot. (A)** A 12% SDS-PAGE gel showing the purified Exs proteins and a MBP-fused Exs protein as positive control for Far-Western Blot. Lane 1, BLUeye Prestained Protein Ladder (Geneflow); Lane 2, purified _His_ExsA protein (**∼**33 kDa); Lane 3, purified _His_ExsC protein (**∼**18.5 kDa); Lane 4, purified _His_ExsD protein (**∼**33 kDa); Lane 5, purified _His_ExsE protein (**∼**11 kDa); Lane 6, purified MBP-ExsC protein (**∼**61 kDa). The proteins on this SDS-PAGE gel were then transferred onto a nitrocellulose membrane for Far-Western Blot. **(B)** Negative control Far-Western Blot of the control protein MBP5 probing His-tagged Exs proteins. Only MBP-ExsC was observed after probing, suggesting that MBP5 protein without Exs fusions was unable to interact with His-tagged Exs proteins. **(C)** Far-Western Blot of MBP-ExsA probing His-tagged Exs proteins. The _His_ExsD protein was detected, suggesting ExsA–ExsD interaction. **(D)** Far-Western Blot of _His_ExsD probing MBP-fused Exs proteins. Only MBP-ExsC (∼61 kDa) was detected when probed with _His_ExsD protein. **(E)** Far-Western Blot of MBP-ExsC probing His-tagged Exs proteins. The _His_ExsD and _His_ExsE proteins were detected, suggesting ExsC–ExsD and ExsC–ExsE interactions. The upper band showing in lane 4 was possibly the size of a dimerized _His_ExsD (**∼**66 kDa). **(F)** Far-Western Blot of MBP-ExsE probing His-tagged Exs proteins. The _His_ExsC and _His_ExsE were detected when probed with MBP-ExsE, suggesting ExsE–ExsC interaction and ExsE–ExsE self-interaction.

The purified four His-tagged Exs proteins were loaded on an SDS-PAGE gel (**Figure 5A**) and blotted together with an MBP-fused ExsC as a positive control. When probed with the MBP5 kit control protein (without any Exs fusion) only the positive control MBP-ExsC fusion protein (and degradation products) was detected, demonstrating that the His-tagged Exs proteins were unable to interact with the MBP5 negative control (**Figure 5B**).

Only the _His_ExsD protein was detected in the Far-Western Blot when probed with MBP-ExsA (**Figure 5C**) indicating the interaction between ExsA and ExsD. No interaction with ExsC or ExsE was seen. Furthermore, no self-interaction with ExsA was observed (**Figure 5C**), contrary to the findings of the BACTH assay.

When MBP-ExsD was used to probe the His-tagged Exs proteins, no bands were observed except for the positive control (data not shown). Therefore, the Far-Western Blot was carried out in reverse, using _His_ExsD to probe the MBP-fused Exs proteins. Only the MBP-ExsC protein was detected (and the positive control), showing the interaction between ExsD and ExsC (**Figure 5D**).

When MBP-ExsC was used as a probe, both the _His_ExsD and _His_ExsE proteins were detected (**Figure 5E**). There were two bands of different sizes detected for _His_ExsD, one was the size of the _His_ExsD (**∼**33 kDa) while the other one was possibly the size of dimerized _His_ExsD (**∼**66 kDa). This demonstrates ExsC–ExsD interaction and ExsC–ExsE interaction. No interaction was seen for ExsA or self-interaction with ExsC (**Figure 5E**).

The _His_ExsC and the _His_ExsE proteins were detected when MBP-ExsE was used as a probe (**Figure 5F**) demonstrating interactions between ExsE and ExsC as well as the ExsE–ExsE self-interaction.

### Mutations in the T3SS Regulatory System Affect Swarming Motility

As the results presented here suggest the presence of a T3SS regulatory cascade of Exs proteins in *A. hydrophila*, further investigation of the effect of the *exs* mutations on swarming motility in *A. hydrophila* were carried out. Different strains of *A. hydrophila* including the *exsA* mutant, *exsC* mutant, *exsD* mutant, *exsE* mutant, and a *lafK* mutant were plated on the swarming agar together with the wild type. The wild type strain was able to swarm on the surface of the 0.6% (w/v) semi-solid agar, but when the major regulator of the *Aeromonas* lateral flagella system was knocked out in the *lafK* mutant, swarming motility was completely lost. Moreover, the swarming motility was not affected in the *exsA* mutant or *exsC* mutant as the mutant strains were able to swarm as much as in wild type strain. However, when the *exsD* gene or *exsE* gene were mutated, the swarming motility was reduced.

**FIGURE 6:**
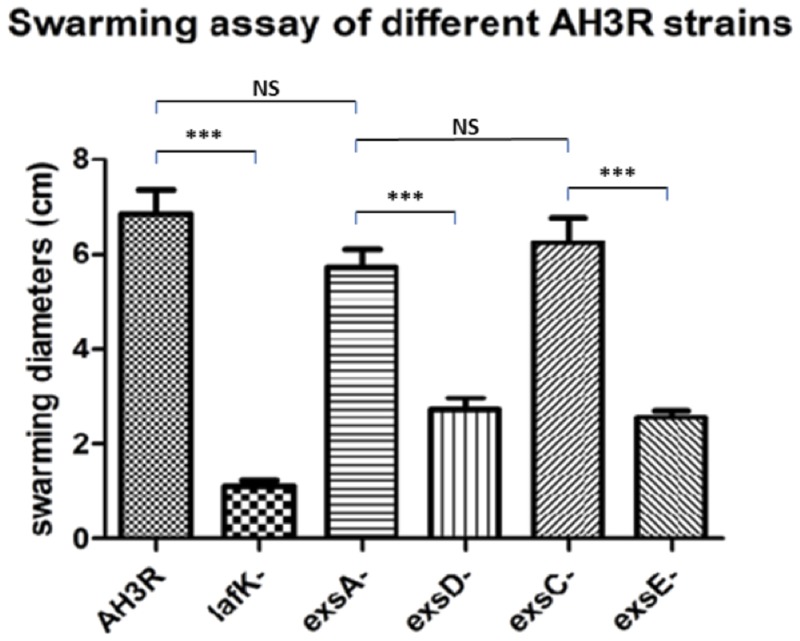
**Swarming assays of different mutant strains of *A. hydrophila* AH3R.** Bacteria were incubated on the swarming agar 0.6% (w/v) at 30°C overnight and the diameter of the swarm measured. The experiment was repeated at least 15 times. Quantification of the swarming assays of *A. hydrophila* AH3R wild type and mutant strains. The swarming diameters were significantly lower in the *lafK* mutant when compared to the *A. hydrophila* AH3R wild type (^∗∗∗^*p* < 0.001), while there was no significant difference of swarming diameters among the wild type strain, the *exsA* mutant and *exsC* mutant (*p* > 0.05). However, the swarming diameters of *exsD* mutant and *exsE* mutant were significant decreased when compared to the *exsA* mutant and *exsC* mutant (^∗∗∗^*p* < 0.001). The error bars showed Standard Error of the Mean (SEM). The significance was determined using Student’s *t*-test.

The phenotypes of the swarming assays were quantified by measuring the swarming diameters of each strain (**Figure 6**). The average swarming diameter of the *A. hydrophila* wild type strain was 6.9 cm while it was significantly decreased to 1.1 cm in the *lafK* mutant strain (*p* < 0.001). The swarming motility of the *exsA* and *exsC* mutant strains was not significantly different from the wild type, with an average swarming diameter of 5.7 and 6.3 cm respectively (*p* > 0.05). Swarming motility was repressed in *exsD* and *exsE* mutant strains when compared to the wild type, and the *exsA* and *exsC* mutant strains (*p* < 0.001). The average swarming diameters of *exsD* mutant and *exsE* mutant strains were 2.7 and 2.6 cm respectively, although reduced these were also significantly different from the *lafK* mutant (*p* < 0.05) (**Figure 6**).

### Exs Regulatory Proteins Modulate Lateral Flagella Promoter Activity

In order to investigate the effect of the mutations on the aeromonad lateral flagella system, several of the lateral flagella promoter regions (Wilhelms et al., 2013) were fused to *lacZ* in the promoter probe transcriptional fusion vector pKAGb-2(-) and introduced into *A. hydrophila* strains. The activities of the lateral flagella promoters were measured in bacteria grown on swarming agar.

Among all putative promoters, *PfliM, PflgB* and *PlafA* were chosen, one from each class of the lateral flagella transcriptional hierarchy (Wilhelms et al., 2013). To investigate the potential cross-talk between the lateral flagella system and the T3SS, promoter activities were measured in different mutant backgrounds.

**FIGURE 7:**
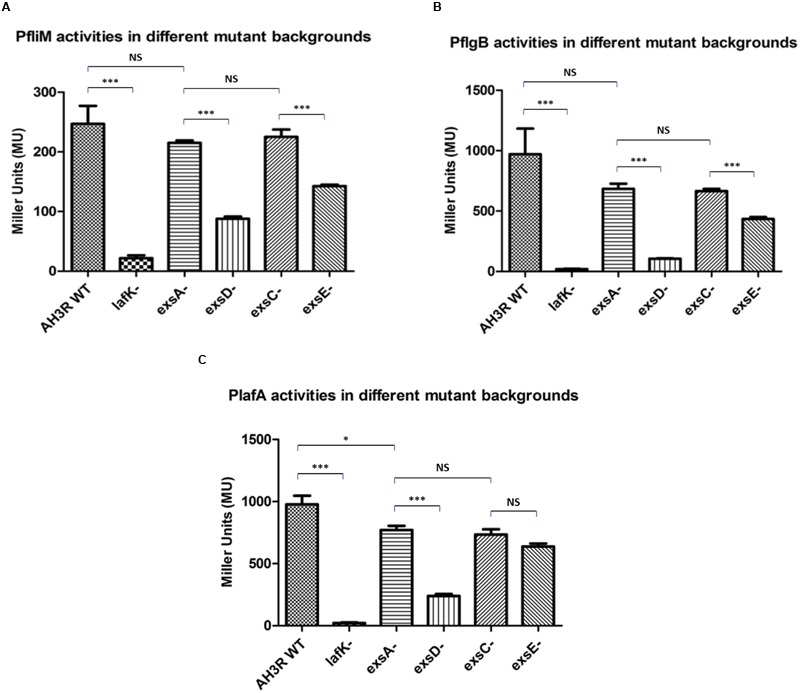
**β-galactosidase activities of the aeromonad lateral flagella promoters *PfliM, PflgB* and *PlafA* in *A. hydrophila* AH3R wild type and mutant backgrounds. (A)** Promoter activities of *PfliM* were significantly decreased in the *lafK* mutant, *exsD* mutant and *exsE* mutant when compared to the AH3R wild type, *exsA* mutant and *exsC* mutant. **(B)** Promoter activities of *PflgB* were significantly decreased in the *lafK* mutant, *exsD* mutant and *exsE* mutant when compared to the AH3R wild type, *exsA* mutant and *exsC* mutant. **(C)** Promoter activities of *PlafA* were significantly decreased in the *lafK* mutant and *exsD* mutant when compared to the AH3R wild type, *exsA* mutant and *exsC* mutant. While in the *exsE* mutant, promoter activity of *PlafA* was significantly lower than in the *exsA* mutant but at a similar level with the *exsC* mutant. The promoter activities were measured when each strain was grown on swarming agar at 30°C overnight. Assays were carried out in triplicate and the values are the means. The error bars showed Standard Error of the Mean (SEM). The significance was determined using Student’s *t*-test (^∗^*p* < 0.05, ^∗∗∗^*p* < 0.001).

The *PfliM* promoter activity in the wild type was 247 MU this decreased to 22 MU in the *lafK* mutant (*p* < 0.001). The promoter activity of *PfliM* was 215 MU in the *exsA* mutant but decreased to 88 MU in the *exsD* mutant strain (*p* < 0.001). Similarly, in the *exsC* mutant strain, the promoter activity of *PfliM* was 225 MU and decreased to 143 MU in the *exsE* mutant strain (*p* < 0.001). There was no significant difference of the promoter activities among the *exsA* mutant, the *exsC* mutant and the wild type strains (*p* > 0.05). However, the promoter activity in the *exsD* mutant was significantly higher than in the *lafK* mutant while it was significantly lower than in the *exsE* mutant (*p* < 0.001) (**Figure 7A**).

The *PflgB* promoter activity was 970 MU in the wild type and decreased to 20 MU in the *lafK* mutant (*p* < 0.001). The promoter activity was 684 MU in the *exsA* mutant strain, which was not significantly different with the promoter activities in the wild type and the *exsC* mutant strain (*p* > 0.05) while it was significantly higher than the promoter activities in the *lafK* mutant, *exsD* mutant and *exsE* mutant strains (*p* < 0.001). The promoter activity of *PflgB* was 105 MU in the *exsD* mutant while it increased to 665 MU in the *exsC* mutant strain (*p* < 0.001). *PflgB* promoter activity was 435 MU in the *exsE* mutant, which was significantly lower than in the wild type, the *exsA* mutant and the *exsC* mutant strains but significantly higher than in the *lafK* mutant and *exsD* mutant strains (*p* < 0.001) (**Figure 7B**).

The pattern of *PlafA* promoter activity was similar to *PfliM* and *PflgB*. In the wild type, *PlafA* was 976 MU that decreased to 22 MU in the *lafK* mutant strain (**Figure 7C**). *PlafA* promoter activity in the *exsA* mutant was 771 MU, which was significantly higher than in the *lafK* mutant (*p* < 0.001), the *exsD* mutant (*p* < 0.001) and the *exsE* mutant (*p* < 0.01). The promoter activity in the *exsD* mutant was 240 MU, which was approximately fourfold lower than in the wild type strain and threefold lower than in the *exsA* mutant strain. *PlafA* activity was 734 MU in the *exsC* mutant strain, which was not significantly different with the promoter activity in the *exsA* mutant (*p* > 0.05). Unlike *PfliM* and *PflgB*, the promoter activity of *PlafA* in the *exsE* mutant strain was 638 MU, which was not different with the promoter activity measured in the *exsC* mutant (p > 0.05) but significantly lower than the *exsA* mutant (*p* < 0.01). The promoter activity of *PlafA* in the *exsE* mutant was also significantly higher than in the *exsD* mutant (*p* < 0.001) (**Figure 7C**).

## Discussion

While screening for non-swarming transposon mutants, two were isolated that had a reduction in swarming motility. These were surprisingly mutated in a gene encoding ExsD a protein putatively involved in the regulation of the aeromonad T3SS. This was similar to the results of Yu et al. (2007), who demonstrated that *exsD* mutants have an effect on aeromonad lateral flagella expression. As little is known about the regulation of the T3SS in *Aeromonas*, this prompted us to further investigate the regulation of this system and its possible cross-talk with the lateral flagella system. The results of this study suggest that the regulatory cascade of the T3SS which involves the ExsA, ExsD, ExsC, and ExsE proteins in *A. hydrophila* is similar to the T3SS regulatory cascade reported in *P. aeruginosa* (Yahr et al., 1995; Diaz et al., 2011). The AraC family transcriptional activator ExsA functions as the master regulator of the T3SS in *A. hydrophila*, as its mutation reduces expression from all the T3SS promoters with the exception of its own. The anti-activator protein ExsD inhibits the master regulator ExsA through direct protein–protein interactions. The chaperone protein ExsC is able to sequester the anti-activator ExsD from ExsA by direct binding to ExsD. While the effector protein ExsE binds to its cognate chaperone protein ExsC directly thus releasing ExsD to inhibit ExsA. The Exs regulatory cascade is also shown to be involved in the regulation of the lateral flagella system, in which ExsA possibly functions in down-regulating the lateral flagella promoter activities and suppressing the swarming motility.

Before measuring the activities of *A. hydrophila* T3SS promoters in the mutant backgrounds, the assay conditions were optimized, as there was evidence showing that the expression of the *A. hydrophila* T3SS was induced significantly with additional 20 mM MgCl_2_ and 10 mM EGTA. Vilches et al. (2009) demonstrated that the expression level of two T3SS mediated genes *aopN* and *aexT* were up-regulated with additional MgCl_2_ and EGTA using *gfp*-fusions. However, in this study, the promoter activities of *PaopN* and *PexsA* were decreased in these conditions. Moreover, no significant difference of promoter activity between the inducing conditions and non-inducing conditions was found for promoters *PexsC* and *PexsD* (data not shown). Similar results were reported by Yu et al. (2007).

In this study, as the T3SS promoters were cloned in the multi-copy *lacZ*-fusion plasmid pKAGb-2(-), this could possibly give rise to a transcriptional factor titration effect. The potential repressors might be ‘outnumbered’ by the high copy number of the promoter (Guido et al., 2006; Brewster et al., 2014). Although pKAGb-2(-) is not a high copy number vector.

In the Vilches’ study, the activity of the *PaopN* promoter was obtained by measuring the expression level of *gfp*-fusion. The *gfp* reporter gene was integrated into the *A. hydrophila* AH-3 chromosomal DNA downstream of the *PaopN* promoter and upstream of the *aopN* gene. The integration of *gfp* gene might affect the expression of the downstream operon, in which at least three genes, *acrR, acrG* and *acrV* were present all believed to be involved in the low calcium response (Barve and Straley, 1990; Matson and Nilles, 2001; Burr et al., 2003).

Five promoter sequences, *PascN, PaopN, PexsC, PexsA* and *PexsD* were investigated in the T3SS regulon and four of them were shown to have no activity in the absence of ExsA, except for *PexsA*, which was responsible for the expression of the master regulator ExsA itself. The promoter activity of *PexsA* was up-regulated in the *exsA* mutant while in the *E. coli* reconstitution system the *PexsA* promoter activity was down-regulated with *exsA in-trans*. This finding indicates that the master regulator ExsA is under control of negative feedback by inhibiting its own promoter. AraC was reported to repress the expression of its own gene *araC* by binding to its own promoter region (Casadaban, 1976; Hahn and Schleif, 1983). As a member of AraC family proteins, there has been no report of ExsA that it can repress its own transcription so far. However, many members of the AraC repress their own expression, such as XylR, one of the regulators for xylene metabolism in *Pseudomonas putida* and YbtA, a pesticin receptor regulator in *Yersinia pestis* (Inouye et al., 1987; Fetherston et al., 1996).

Due to the lack of T3SS in *E. coli* re-constitution system, of the T3SS promoters, except for *PexsA*, had low promoter activity, suggesting that these promoters required certain aeromonad or T3SS-specific factors other than ExsA in order for optimal activation. This contributed to the previous suspicion that there might be secondary regulation or regulators other than the master regulator ExsA. The promoter *PexsA* was still highly active in *E. coli* in the absence of *exsA* in-trans, thus the promoter was likely to be activated by housekeeping sigma factor σ^70^ in *E. coli*. While in *E. coli* with induced *exsA* expression, the promoter activity of *PexsA* was down-regulated significantly. This confirmed that ExsA negatively regulates its own promoter PexsA. Therefore, *PexsA* is constantly activated but under a negative feedback control of ExsA. Therefore, it could be deduced that the *A. hydrophila* maintains a minimal level of T3SS expression in the environment due to the constitutive expression of ExsA. When bacteria encounter host cells, according to our hypothesis, ExsE is secreted and ExsC is free to bind to ExsD releasing ExsA to further activate the T3SS.

Both the BACTH assay and the Far-Western Blot indicate interactions in between ExsA–ExsD, ExsD–ExsC, and ExsC–ExsE, supporting the hypothesis of the ExsA–ExsD–ExsC–ExsE regulatory cascade. Moreover, the self-interaction of ExsA protein was observed from BACTH assay while the self-interaction of the ExsE protein was observed from Far-Western Blot.

In *P. aeruginosa* the C-terminal domain of ExsA has two helix-turn-helix DNA binding motifs but lacks the ability to self-interact for cooperative binding of DNA (Brutinel et al., 2009). ExsD was shown to inhibit the DNA-binding ability of ExsA by interacting with the N-terminal domain of ExsA, which is involved in the ExsA-ExsA self-interaction (Brutinel et al., 2010). However, in this study, the C-terminal domain of the ExsA protein was shown to interact with both the N-terminal and C-terminal domain versions of itself in the BACTH assay.

When MBP-ExsA was used to probe His-tagged Exs proteins, only His-ExsD was detected although the interaction appeared weak. This correlates with *P. aeruginosa* that ExsD is found to bind ExsA only as a folding intermediate when these two proteins were synthesized together (Bernhards et al., 2013). Thereby, strong interactions between ExsD and ExsA were observed *in vivo* using BACTH assay when they were co-expressed whereas weak interactions were shown *in vitro* using Far-Western Blot.

The self-association of ExsD and ExsC reported in *P. aeruginosa* was not observed in this study (Diaz et al., 2011). As it has been reported in *P. aeruginosa*, ExsC dimerises to form a 2:2 heterotetramer with ExsD while it binds to ExsE at a 2:1 ratio (Zheng et al., 2007; Bernhards et al., 2013). This might be due to the presence of T25/T18, His_6_ or MBP onto the N-terminal regions of these proteins.

The interactions of ExsC with each of the Exs proteins shown in the BACTH assay revealed a potential hierarchy of ExsC interactions. The potential strongest interaction was observed in ExsC–ExsE, which was a typical T3SS chaperone–effector interaction described in *P. aeruginosa* (Dasgupta et al., 2004; Urbanowski et al., 2005; Vogelaar et al., 2010). Although in this study the no binding kinetics were carried out, the ExsC–ExsD interaction was thought to be weaker than the ExsC–ExsE interaction but stronger than the ExsC–ExsC self-interaction from the observation of the BACTH assay. This finding corresponds to the Isothermal Titration Calorimetry studies with binding affinities for ExsC–ExsD (18 nM) and ExsC–ExsE (1 nM) in *P. aeruginosa* (Zheng et al., 2007). This suggests that the chaperone protein ExsC prefers to bind to the effector protein ExsE rather than the anti-activator ExsD. However, when the concentration of ExsE decreases, the abundant ExsC proteins bind to ExsD and antagonize the inhibition of ExsD on ExsA.

The swarming assay of the *exs* mutants has shown reduced swarming motility in *exsD* mutant and *exsE* mutant strains similar to the *lafK* mutant strain. It suggests that the T3SS master regulator ExsA functions directly or indirectly in repressing the expression of the lateral flagella system in *A. hydrophila* AH-3. It correlates with the findings in *Vibrio parahaemolyticus*, which possess lateral flagella system as well as two T3SSs. The expression level of the lateral flagella gene *flgB_L_* was significantly repressed when over-expressing ExsA and the swarming motility of *V. parahaemolyticus* was inhibited by *exsA* induction *in vivo* (Gode-Potratz et al., 2010).

In *V. parahaemolyticus* and *A. hydrophila* the lateral flagella system is hierarchically controlled at three levels (Canals et al., 2006; Wilhelms et al., 2013).

One lateral flagella promoter was chosen from Class I (*PfliM*), Class II (*PflgB*) and Class III (*PlafA*) and the promoter activities were measured in the wild type, *lafK* and *exs* mutant strains in order to investigate the potential cross-talk between the lateral flagella system and the T3SS. The patterns of the promoter activities (*PfliM, PflgB* and *PlafA*) measured in the *exs* mutant backgrounds were similar to the patterns of the swarming diameters of different mutant strains. It indicates that the lateral flagella promoter activities are significantly repressed in the absence of ExsD and ExsE, while not affected in the absence of ExsA and ExsC, supporting the hypothesis that the T3SS master regulator ExsA functions as a repressor of the lateral flagella system. When the anti-activator ExsD is absent, the abundant ExsA proteins suppress the expression of the lateral flagella system. Similarly, when ExsE is absent, the chaperone protein ExsC binds to the anti-activator ExsD thus freeing ExsA to repress the lateral flagella system. There was evidence of cross-talk between the T3SS and the flagella system in *P. aeruginosa, Y. enterocolitica* and *A. hydrophila* AH-1 (Bleves et al., 2002; Soscia et al., 2007; Yu et al., 2007; Vilches et al., 2009). Similar negative cross-talk between the T3SS and the flagella system was determined later in *P. aeruginosa* as well (Soscia et al., 2007).

In this study the regulatory components of the T3SS were demonstrated to be involved in the regulation of the lateral flagella system in *A. hydrophila*. Whether the lateral flagella system affects the T3SS in *A. hydrophila* is still unknown, although there is evidence that the lateral flagella regulator LafK is required for T3SS1 expression in *V. parahaemolyticus* (Gode-Potratz et al., 2010).

The overall results correlate with the findings in *V. parahaemolyticus*, which possesses the lateral flagella system and two T3SSs (T3SS1 and T3SS2). It was reported by Gode-Prtratz et al. (2010) that the expression level of the lateral flagella gene *flgBL* was significantly repressed when over-expressing ExsA, and the swarming motility of *V. parahaemolyticus* was inhibited by *exsA* induction *in vivo*.

However, it is still unclear how ExsA represses the expression of the lateral flagella system, since the lateral flagella promoter activities were decreased in the absence of ExsD despite the promoter class. The Class I promoter *PfliM*, the Class II promoter *PflgB* and the Class III promoter *PlafA* were all affected by the absence of ExsD and ExsE. The fact that the Class I promoter is affected suggests that the ExsA protein represses the expression of the lateral flagella system prior to the lateral flagella major regulator LafK.

**FIGURE 8:**
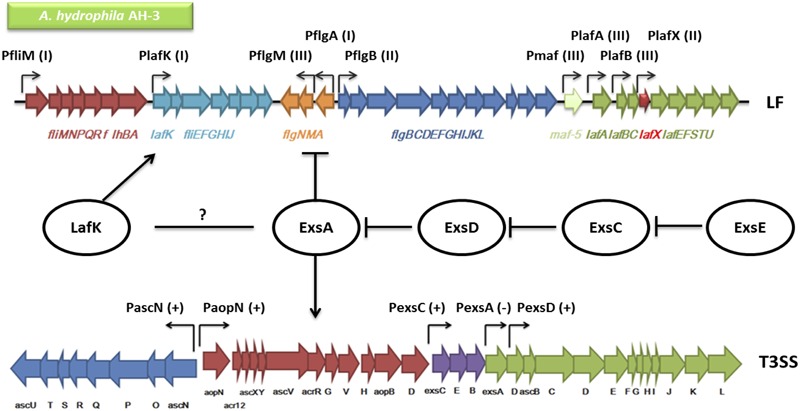
**An overview of the genetic regulation between the T3SS and the lateral flagella (LF) system in *A. hydrophila* AH-3.** The regulators including ExsA, ExsD, ExsC, ExsE, and LafK are shown in circles. Each two of the Exs protein are connected by blunt arrows showing the inhibitory interactions in between. The promoters in both the T3SS and the LF system are shown in bent arrows. The promoters in the T3SS are either (+) up-regulated by ExsA or (-) down-regulated by ExsA (*PaopN* and *PexsC* data not shown). The LF promoters are categorized into (I) Class I, (II) Class II, or (III) Class III, in which only Class II and Class III promoters are up-regulated by the LF major regulator LafK. The T3SS master regulator ExsA has been shown to down-regulate the expression of the LF system but whether there is interaction between ExsA and LafK is unknown (Genetic alignments of the T3SS and LF were adapted from Vilches et al., 2004 and Canals et al., 2006).

A schematic overview of a model of the genetic regulation between the T3SS and the lateral flagella system is shown in **Figure 8**. The T3SS master regulator ExsA activates the transcription of the T3SS by inducing the T3SS promoter activities, except that ExsA negative regulates its own promoter *PexsA*. The master regulator ExsA is controlled by a cascade of proteins including ExsD, ExsC, and ExsE. The anti-activator ExsD inhibits ExsA by direct binding. ExsC inhibits ExsD and ExsE inhibits ExsC via direct protein–protein interactions as well. ExsA was also shown to negatively regulate the lateral flagella system since the absence of ExsD or ExsE represses the swarming ability and the activities of lateral flagellar promoters.

## Author Contributions

All authors have made substantial, direct and intellectual contribution to the work and approved it for publication. Y-HZ performed the experiments and helped write the manuscripts. JS directed the research, had intellectual input into the experimental design and helped write the manuscript.

## Conflict of Interest Statement

The authors declare that the research was conducted in the absence of any commercial or financial relationships that could be construed as a potential conflict of interest.
