# Supplementary material for: Cross-Talk between the Aeromonas hydrophila Type III Secretion System and Lateral Flagella System
Source: Front Microbiol. 2016 Sep 7;7:1434. doi: 10.3389/fmicb.2016.01434 (PMC5013049; doi:10.3389/fmicb.2016.01434)
Supplement: Supplementary file 1 [file Table_1.DOCX]

**Supplementary Table 1. Primers used in this study**

*Tn5-O-End-Ex*: CAAGCTTCGGCCGCCTAGGCCGCG

*Tn5-I-End-Int:* CCAGATCTGATCAAGAGACAGTCT

*exsC* internal forward: CTCCACTTTGGTTTCGATGA

*exsC* internal reverse: ATTGAACTGATACCAGTGAC

*exsC* amplification forward: GGAGGAAATCATGGATGTAA

*exsC* amplification reverse: ATCTTCATGGTTATACCCGC

*exsD*F1forward: GAGCTCGGTACCCGGGGATCCTCTAGAGTCATGAGTCAGCAAGATCACAA

*exsD*F1reverse: AAGCTGTCAAACATGAGAACCAAGGAGAATGATGCGATCTCCCAGCTGT

*exsD*F2forward: GAATTGTTTTAGTACCTAGCCAAGGTGTGCTCGCGCCTTGCTGTGGCACT

*exsD*F2reverse: AGAATACTCAAGCTTGCATGCCTGCAGGTCGCTATCTAGGGCTCGGCAGG

*exsE*F1forward: GAGCTCGGTACCCGGGGATCCTCTAGAGTCTCAATCGGCTGCTCACTGAG

*exsE*F1reverse: AAGCTGTCAAACATGAGAACCAAGGAGAAACTTGGCTCGCACTGGCTGG

*exsE*F2forward: GAATTGTTTTAGTACCTAGCCAAGGTGTGCGCACTTCTGCGACGCTCCAT

*exsE*F2reverse: AGAATACTCAAGCTTGCATGCCTGCAGGTCCAATGTTTGGGCTGCAATTG

Kan forward: TTCTCCTTGGTTCTCATGTTTGACAGCTT

Kan reverse: GCACACCTTGGCTAGGTACTAAAACAATTC

exsD_pGEM forward GCGAATTCATGAGTCACCAAGATCACAA

exsD_pGEM reverse GCGAATTCGCTATCTAGGGCTCGGCAGG

exsE_pGEM forward GCGAATTCTCAATCGGCTGCTCACTGAG

exsE_pGEM reverse GCGAATTCCAATGTTTGGGCTGCAATTG

*exsA*_pKT_forward GCCTCTAGAGATGAATGGCATTACTACTGCAG

*exsA*_pKT_reverse GCGAATTCTTAATCAGTGCCATGTCTGGC

*exsC*_pKT_forward GCTCTAGAGGATGTAACTGTCATCATCAA

*exsC*_pKT_reverse GCGAATTCTTATACCCGCACTCCCATCA

*exsD*_pKT_forward GCGCTCTAGACATGAGTCAGCAAGATCACAATTC

*exsD*_pKT_reverse GAGAATTCCTAGGGCTCGGCAGGCTGCCA

*exsE*_pKT_forward GCTCTAGAGAAGATTCAGGAATCACAAGG

*exsE*_pKT_reverse GCGAATTCTCATAACACCCGGATCCGAC

*exsA*_pKNT_forward GCGCAAGCTTGATGAATGGCATTACTACTGCAG

*exsA*_pKNT_reverse GCTCTAGAGAATCAGTGCCATGTCTGGC

*exsC*_pKNT_forward GCTCTAGAGGATGTAACTGTCATCATCAA

*exsC*_pKNT_reverse GCGAATTCGATACCCGCACTCCCATCACCT

*exsD*_pKNT_forward GCAAGCTTGATGAGTCAGCAAGATCACAATTC

*exsD*_pKNT_reverse GCTCTAGATAGGGCTCGGCAGGCTGCCAGT

*exsE*_pKNT_forward GCTCTAGAGAAGATTCAGGAATCACAAGG

*exsE*_pKNT_reverse GCGAATTCGATAACACCCGGATCCGACGTT

*exsA*_pUT_forward GCGCAAGCTTGATGAATGGCATTACTACTGCAG

*exsA*_pUT_reverse GCTCTAGAGAATCAGTGCCATGTCTGGC

*exsC*_pUT_forward GCTCTAGAGGATGTAACTGTCATCATCAA

*exsC*_pUT_reverse GCGAATTCGATACCCGCACTCCCATCACCT

*exsD*_pUT_forward GCAAGCTTGATGAGTCAGCAAGATCACAATTC

*exsD*_pUT_reverse GCTCTAGATAGGGCTCGGCAGGCTGCCAGT

*exsE*_pUT_forward GCTCTAGAGAAGATTCAGGAATCACAAGG

*exsE*_pUT_reverse GCGAATTCGATAACACCCGGATCCGACGTT

*exsA*_pUT18C_forward GCCTCTAGAGATGAATGGCATTACTACTGCAG

*exsA*_pUT18C_reverse GCGAATTCTTAATCAGTGCCATGTCTGGC

*exsC*_pUT18C_forward GCTCTAGAGGATGTAACTGTCATCATCAA

*exsC*_pUT18C_reverse GCGAATTCTTATACCCGCACTCCCATCA

*exsD*_pUT18C_forward GCGCTCTAGACATGAGTCAGCAAGATCACAATTC

*exsD*_pUT18C_reverse GAGAATTCCTAGGGCTCGGCAGGCTGCCA

*exsE*_pUT18C_forward GCTCTAGAGAAGATTCAGGAATCACAAGG

*exsE*_pUT18C_reverse GCGAATTCTCATAACACCCGGATCCGAC

pKT25_screen forward GCACATGTTCGCCATTATGCCG

pKT25_screen reverse GCATTCAGGCTGCGCAACTGTT

pKNT25_screen forward GCACAGGTTTCCCGACTGGAAA

pKNT25_screen reverse GCCGGAACATCAATGTGGCGTT

pUT18_screen forward GCCACCCCAGGCTTTACACTTT

pUT18_screen reverse GCTCACGCCGATATTCATGTGG

pUT18C_screen forward GCAAAAGCCTGTTCGACGATGG

pUT18C_screen reverse GCTCACAGCTTATCTGTAAGCG

*exsC* for pET forward GCCATATGGATGTAACTGTC

*exsC* for pET reverse GCAAGCTTTTATACCCGCAC

*exsD* for pET forward GCCATATGAGTCAGCAAGAT

*exsD* for pET reverse GCAAGCTTCTAGGGCTCGGC

*exsE* for pET forward GCCATATGAAGATTCAGGAATCACAAGG

*exsE* for pET reverse GCGAATTCTCATAACACCCGGATCCGACG

T7 promoter (forward): TAATACGACTCACTATAGGG

T7 terminator (reverse): GCTAGTTATTGCTCAGCGG

pMAL screening primer forward GGTCGTCAGACTGTCGATGAAGCC

pMAL screening primer reverse TGTCCTACTCAGGAGAGCGTTCAC

*PfliM* amplification forward GCAAGCTTAGATCCATGGCGTCAAGAAG

*PfliM* amplification reverse GCGGATCCCAAAGTAACCGAGAAGGTGT

*PlafK* amplification forward GCAAGCTTTGGATAGCTTTCCGGTTGAT

*PlafK* amplification reverse GCGGATCCCGCAGGTCTGATCAATAACA

*PflgM* amplification forward GCAAGCTTCGCAGTGACCCATTTGCCAC

*PflgM* amplification reverse GCGGATCCCTGCAGCTGGGTTTGAACAT

*PflgA* amplification forward GCAAGCTTGCAGTATTGCCATCCATGGA

*PflgA* amplification reverse GCGGATCCGGGAAACAACACTTCCCCTT

*PflgB* amplification forward GCAAGCTTTTCTCGACGAGATCTCCGGT

*PflgB* amplification reverse GCGGATCCTAATCGACATCTCGGGCCAG

*Pmaf* amplification forward GCAAGCTTTACAGTTCAGAGCGACTCGA

*Pmaf* amplification reverse GCGGATCCGGTACGCTGTTGACATCCTG

*PlafA* amplification forward GCAAGCTTGGAGCTCTATATCAAGACCC

*PlafA* amplification reverse GCGGATCCCCAGCATCTTGTTGGTAGAG

*PlafB* amplification forward GCAAGCTTTTCGATGCATCCACCAAAGT

*PlafB* amplification reverse GCGGATCCCTCTGCTGACCTTTGATGCC

*PlafX* amplification forward GCAAGCTTAGGCCATATTCTTGCCAAGC

*PlafX* amplification reverse GCGGATCCGTTGCAAGATCAGACGACCC

pKAGb2(-) screening primer *bla* (forward) TGCACCCAACTGATCTTCAG

pKAGb2(-) screening primer *lacZ* (reverse) TTTCCCAGTCACGACGTTGT
